# Trocar-site hernia following laparoscopic salpingo-oophorectomy in a middle-aged Japanese woman: an initial case report after 40 years of experience at a single center and a brief literature review

**DOI:** 10.1186/s12905-021-01528-6

**Published:** 2022-01-08

**Authors:** Kuniaki Ota, Yukiko Katagiri, Masafumi Katakura, Takafumi Mukai, Kentaro Nakaoka, Toshimitsu Maemura, Toshifumi Takahashi, Mineto Morita

**Affiliations:** 1 Department of Obstetrics and Gynecology, Toho University, 6-11-1 Omori-Nishi, Ota-ku, Tokyo, 143-8751 Japan; 2 Fukushima Medical Center for Children and Women, Fukushima Medical University, 1 Hikarigaoka, Fukushima-shi, Fukushima, 960-1295 Japan

**Keywords:** Trocar site hernia, Laparoscopy, Salpingo-oophorectomy, Minimally invasive surgery

## Abstract

**Background:**

In gynecology, the number of laparoscopic surgeries performed has increased annually because laparoscopic surgery presents a greater number of advantages from a cosmetic perspective and allows for a less invasive approach than laparotomy. Trocar site hernia (TSH) is a unique complication that causes severe small bowel obstruction and requires emergency surgery. Its use has mainly been reported with respect to gastrointestinal laparoscopy, such as for cholecystectomy. Contrastingly, there have been few reports on gynecologic laparoscopy because common laparoscopic surgeries, such as laparoscopic salpingo-oophorectomy, are considered low risk due to shorter operative times. In this study, we report on a case of a woman who developed a TSH 5 days postoperatively following a minimally invasive laparoscopic surgery that was completed in 34 min.

**Case presentation:**

A 41-year-old woman who had undergone laparoscopic salpingo-oophorectomy 5 days previously presented with the following features of intestinal obstruction: persistent abdominal pain, vomiting, and inability to pass stool or flatus. A computed tomography scan of her abdomen demonstrated a collapsed small bowel loop that was protruding through the lateral 12-mm port. Emergency surgery confirmed the diagnosis of TSH. The herniated bowel loop was gently replaced onto the pelvic floor and the patient did not require bowel resection. After the surgical procedure, the fascial defect at the lateral port site was closed using 2-0 Vicryl sutures. On the tenth postoperative day, the patient was discharged with no symptom recurrence.

**Conclusions:**

The TSH initially presented following laparoscopic salpingo-oophorectomy; however, the patient did not have common risk factors such as obesity, older age, wound infection, diabetes, and prolonged operative time. There was a possibility that the TSH was caused by excessive manipulation during the tissue removal through the lateral 12-mm port. Thereafter, the peritoneum around the lateral 12-mm port was closed to prevent the hernia, although a consensus around the approach to closure of the port site fascia had not yet been reached. This case demonstrated that significant attention should be paid to the possibility of patients developing TSH. This will ensure the prevention of severe problems through early detection and treatment.

**Supplementary Information:**

The online version contains supplementary material available at 10.1186/s12905-021-01528-6.

## Background

In recent years, laparoscopic surgery has become increasingly common. It has several advantages when compared to laparotomy, such as faster recovery times, shorter hospital stays, less tissue damage, less bleeding, less pain, and no large incisions. As women typically prefer the cosmetic and non-invasive advantages of laparoscopic surgery, the number of gynecologic laparoscopic surgeries that have been performed in Japan has increased annually [1]. The advantages of laparoscopic surgery are well established; however, it carries its own unique risks and complications, such as the development of a trocar site hernia (TSH). Studies have described incidents of TSH, which can cause severe small bowel obstruction and require emergency surgery as treatment, in cases that followed laparoscopic digestive surgery. Based on the largest available studies, estimates of the incidence of laparoscopic TSH across all surgical subspecialties range from 0.2 to 1.3% [2–5].

Despite the fact that TSH was first reported by Fear et al., who were gynecologists, in 1968 [6], reports on cases of TSH following gynecologic laparoscopic surgery have been sparse. There are few reports of TSH after ovarian tumor surgery as most patients are young and the surgeries involve short operative times and a small number of surgical ports [5, 7, 8]. However, several risk factors for the development of TSH have been proposed, such as older age, diabetes mellitus, prolonged operative times, incision site enlargement, and multiple trocar insertions [9, 10]. In the 40 years of experience at our single center, this was the first case of TSH that occurred 5 days after laparoscopic adnexal surgery, which was not previously identified as a risk factor for TSH. This study was reported in accordance with the CARE guidelines.

## Case presentation

**Fig. 1:**
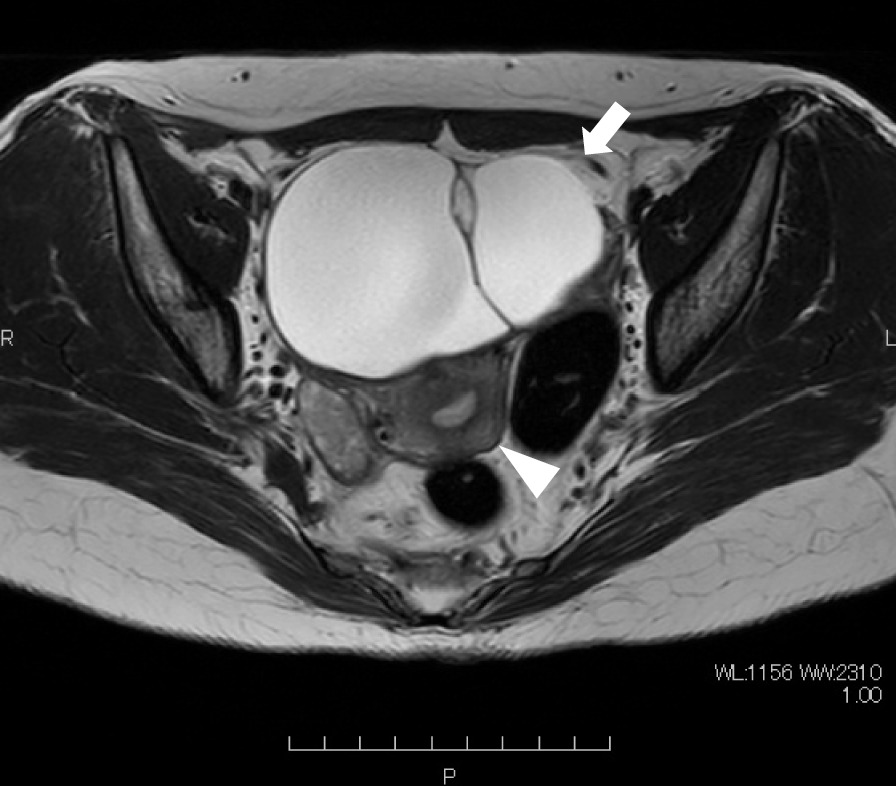
Axial T2-weighted magnetic resonance image of the adnexal mass with multiple cystic cavities in the left adnexal region (arrow). The mass was identified as adjacent to the uterus with the endometrium (arrowhead)

**Fig. 2:**
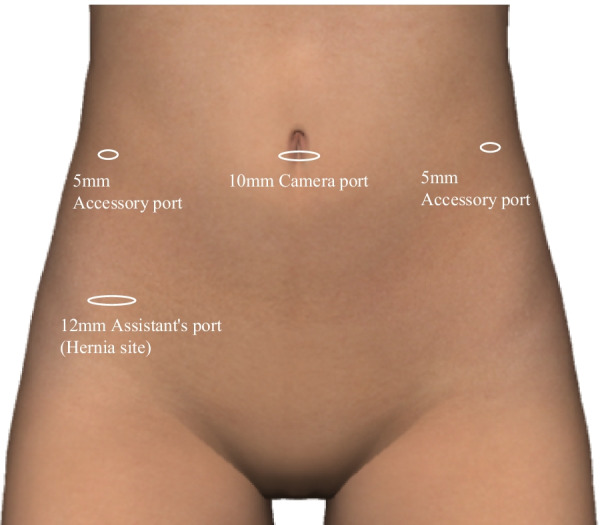
Trocar placement for laparoscopy

**Fig. 3:**
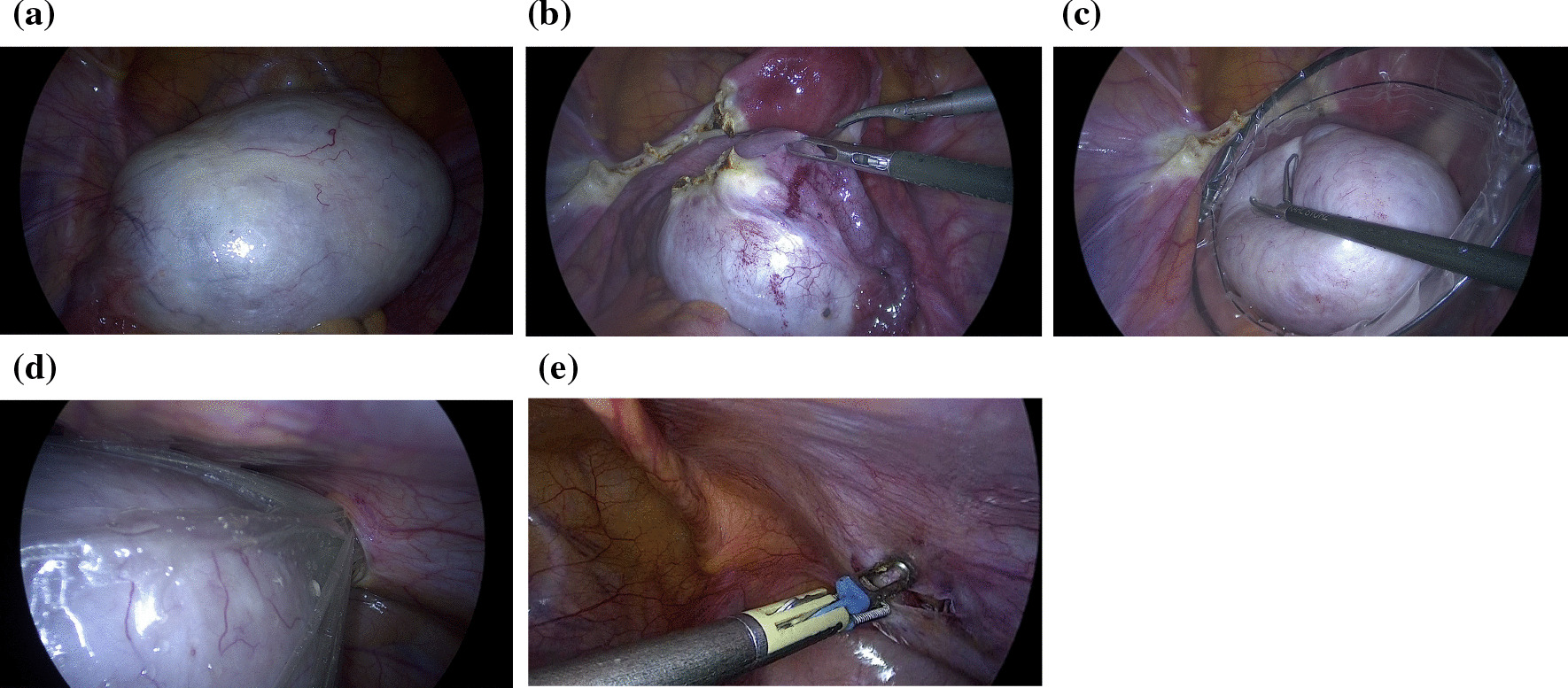
Laparoscopic view. **a** An oversized ovarian cyst was identified in the pelvic cavity. **b**, **c** Salpingo-oophorectomy was performed and the mass was retrieved in an endobag. **d** The excised tissue was extracted through the 12-mm trocar port. **e** After removal of the 12-mm port, the peritoneum was coagulated using bipolar forceps to ensure adequate peritoneal closure

The patient was a 41-year-old, nulliparous woman with a body mass index (BMI) of 21.4 kg/m^2^. She had no known medical history and no history of abdominal surgery. A left-sided, multilocular ovarian cyst that measured 10 cm in diameter was detected on transvaginal ultrasonography, and the patient’s cancer antigen 125 was 13.6 U/ml. Pelvic magnetic resonance imaging revealed a high-signal, left adnexal mass that measured 10 cm in diameter on the T2-weighted images (Fig. 1). Preoperatively, the patient was diagnosed with a mucinous left ovarian tumor and a laparoscopic left salpingo-oophorectomy under general anesthesia was scheduled. The Veress needle technique was used, and a 10-mm umbilical port was introduced using a 10-mm bladed trocar (Karl Storz SE & Co. KG, Tuttlingen, Germany). The patient’s abdomen was insufflated with carbon dioxide (CO_2_) to a pressure of 10 mmHg. The following three accessory port techniques were used: two 5-mm bladed trocars (Karl Storz SE & Co. KG, Tuttlingen, Germany) were inserted into the patient’s lumbar region bilaterally and the third port was inserted into the lower abdominal quadrant, lateral to the right inferior epigastric artery using a 10-mm bladeless trocar (VersaOne™ Bladeless Optical Trocar: Medtronic, Minneapolis, MN, United States of America [USA]; Fig. 2 shows the port placements). All of the ports were successfully inserted on the first attempt, with one trocar pass for each port. None of the port sites were stretched during the procedure. During the laparoscopic surgery, a left ovarian cyst was detected with no adhesions to the abdominal cavity (Fig. 3a). The left adnexa, including the ovarian cyst, was excised using an ultrasonic scalpel (Harmonic Scalpel; Ethicon Endo-Surgery, Cincinnati, OH, USA) and retrieved using an endobag (Endopouch Retriever; Ethicon Endo-Surgery, Cincinnati, OH, USA) through the right lateral 12-mm port (Fig. 3b–d). The excision caused no further elongation of the wound. After the procedure was completed, the peritoneum was closed and coagulated using bipolar forceps to ensure adequate peritoneal closure under direct vision (Fig. 3e). The umbilical port was removed after complete evacuation of the CO_2_. The skin over the umbilical and lateral ports was closed using interrupted sutures and 4–0 polydioxanone (PDS). The operative time was 34 min and there was minimal blood loss. The immediate postoperative course was uneventful, and the patient was discharged on the third postoperative day.

**Fig. 4:**
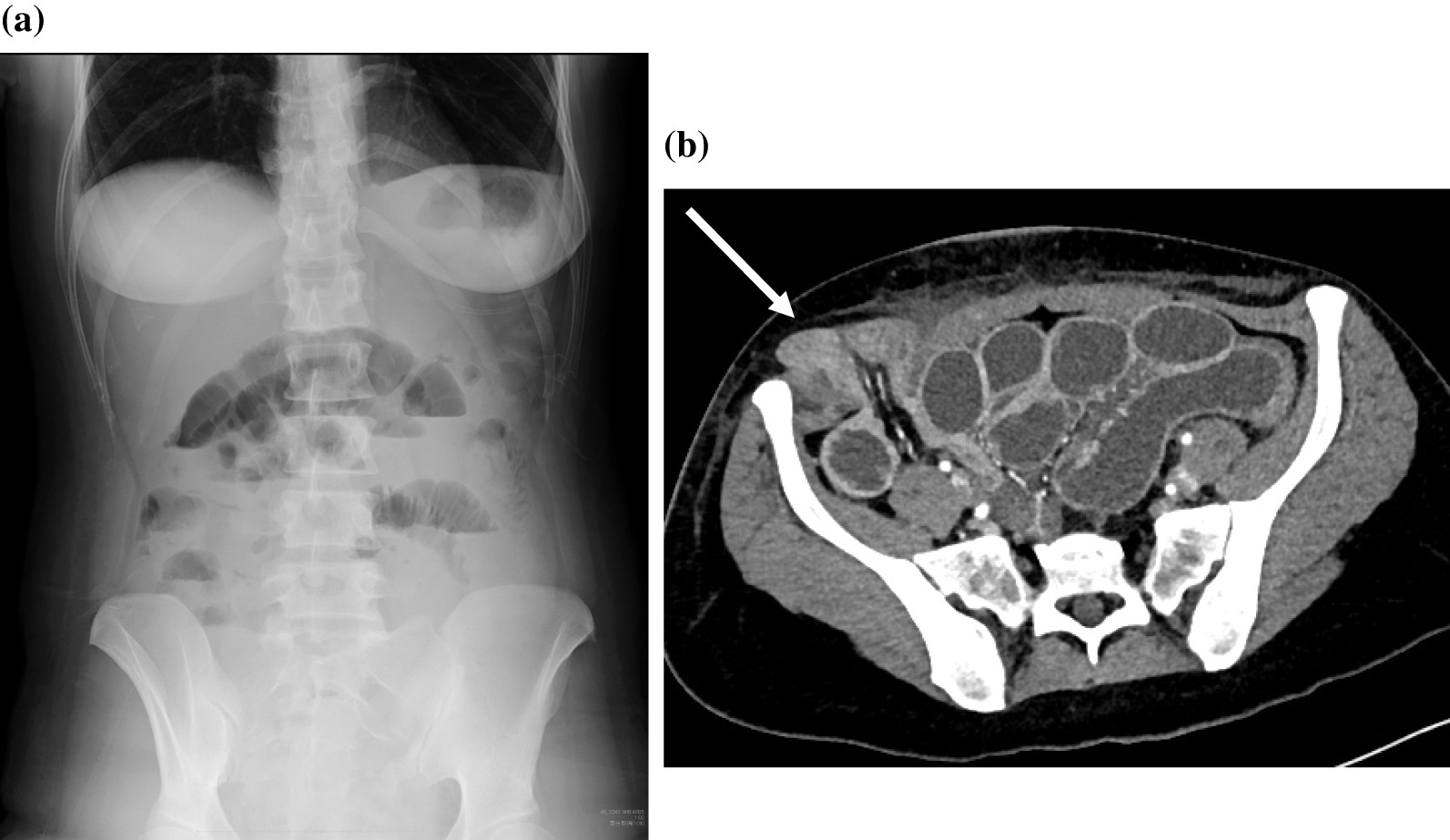
**a** The abdominal X-ray demonstrated multiple air-fluid levels, suggesting small bowel obstruction. **b** The abdominal contrast enhanced computed tomography scan of the small bowel loops above the fascia in the patient with herniation through a 12-mm left lateral trocar site (arrow)

On the fifth postoperative day, she presented to the emergency department with the following symptoms of intestinal obstruction: persistent abdominal pain, vomiting, and inability to pass stool or flatus. Her general condition was average, and she was conscious. Taking the patient's complaints into account, we performed an abdominal X-ray on the suspicion that the patient had developed ileus (Fig. 4a). An abdominal computed tomography scan was performed after the appearance of air-fluid levels on the scan, and a herniated loop of small bowel was detected under the skin from a defect that was created by the right lateral 12-mm trocar entry site (Fig. 4b). The patient exhibited symptoms of intestinal obstruction and was subsequently diagnosed with a strangulated hernia.

**Fig. 5:**
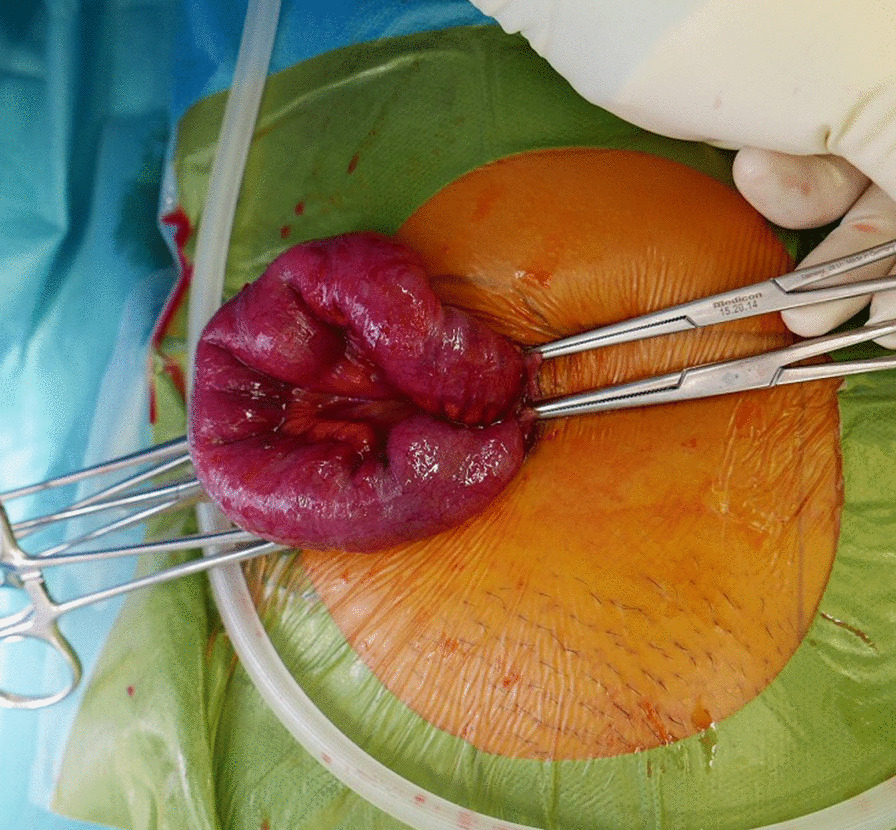
Exploratory laparotomy revealed that a segment of congested small-bowel loop had herniated through the right lateral trocar site

The general surgical and gynecological teams collaborated to perform the operation. The previous right lateral incision was reopened using blunt dissection, and the surgeons discovered that the small bowel loop had herniated directly through the right lateral port (Fig. 5). The right lateral port site was enlarged to 5 cm externally. A finger was used to gently reduce the herniated bowel through the fascial defect, while downward pressure was applied from the skin incision on the right side. The herniated bowel loop appeared dusky following the reduction; however, it was still well perfused and viable after irrigation with warm saline and observation for 20 min. The rest of the bowel and pelvis appeared normal. As the herniated loop was not necrotic, bowel resection was deemed unnecessary. The fascial port sites were closed using 2-0 polyglactin (Vicryl) for the sheath and 3-0 polyglactin for the skin. The total operative time was 1 h and 23 min with minimal bleeding. The patient recovered well postoperatively. Her initial symptoms resolved, and she was discharged 10 days postoperatively with normal bowel function.

## Discussion and conclusions

**Fig. 6:**
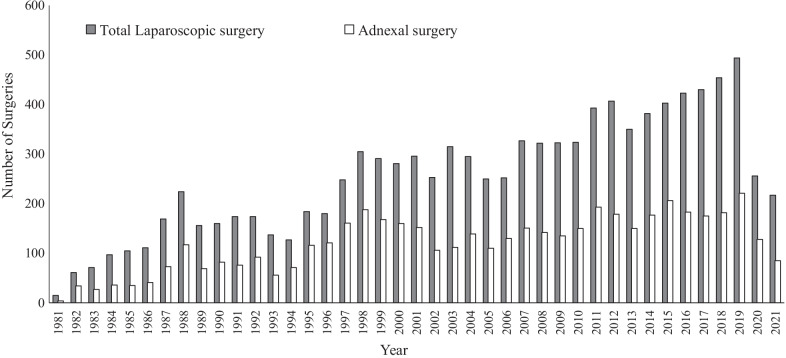
The total number of gynecologic laparoscopies performed each year at Toho University Hospital

This was our first experience with TSH following laparoscopic salpingo-oophorectomy, although we have performed approximately 10,436 laparoscopic surgeries in 40 years, including approximately 4,933 adnexal surgeries since 1981 (Fig. 6). In 1994, the American Association of Gynecologic Laparoscopists published a large-scale study of 4,385,000 patients that determined that the incidence of TSH was 0.021% [11]. Thus, TSH is a rare postoperative complication of gynecologic laparoscopic surgery. Although our patient was able to undergo minimally invasive hernia repair through extension of the 12-mm port incision following the TSH, which is a rare event, life-threatening complications such as bowel obstruction and strangulation can cause significant morbidity and mortality if not addressed promptly [12].

The risk of TSH increases relative to specific patient factors, such as obesity, older age, wound infection, diabetes, and smoking, and surgery-related factors, such as the operative time, excessive trocar insertion port manipulation, port diameter, insertion site, and incomplete suturing of the fascia and peritoneum [13–16]. A previous systematic review recommended closure of all fascial defects > 10 mm and closure of defects > 5 mm when the ports were subjected to excessive manipulation [12]. Contrastingly, one study reported a 0% TSH incidence rate with 5-mm and 10-mm port sites after 4.94 years of follow-up [17]. Another study demonstrated a 0% TSH incidence rate with 10-mm ports, suggesting that fascial closure was unnecessary [18]. In a recent review, Guiterrez et al. concluded that there was no difference in TSH rates if the fascia was left open or closed with ports < 5 mm or > 10 mm [19]. In contrast, the trocar location may be a risk factor for TSH. Due to the inherent anatomical weakness of the paraumbilical region, off-midline trocars had lower TSH incidence rates than midline trocars [4, 19]. In addition, even without fascial closure, non-bladed trocars were associated with decreased bowel obstruction and hernia formation [20, 21]. Guiterrez et al. also established that the use of non-bladed trocars, similar to those used in our method, was associated with lower TSH rates when compared to bladed trocars. Based on the aforementioned evidence, we used non-bladed trocars with a 12-mm port in an off-midline location to prevent TSH.

Female sex tended to be associated with a raised TSH incidence [3, 22, 23]. In particular, older women may be predisposed to TSH due to weaker fascia and a less muscular abdominal wall compared to older men [24]. Furthermore, a high BMI may also be a risk factor for TSH due to increased intra-abdominal pressure [25] and the difficulties around achieving full-thickness closure [26]. In this case, none of the aforementioned risk factors were identified, as the patient had a low BMI and was a middle-aged woman. As previously discussed, although fascial closure had not been performed in the preceding 40 years, in this case, we could not preoperatively predict the TSH occurrence and, fortunately, the TSH could be diagnosed quickly.

A recent, large, retrospective study reported that the total rate of postoperative TSH among gynecologic laparoscopy procedures in the last 20 years was approximately 0.016% (9/55,244) [7]. The study established that the TSH rate was 4/31,778 (0.013%) in laparoscopic salpingo-oophorectomy. This finding was similar to that of our study. However, three of the four cases placed the trocar in the midline, and two of the four cases involved single-incision laparoscopic surgery (SILS). SILS is a known risk factor for TSH [27, 28] because the ports are introduced through a single 2–4 cm incision that is usually in the umbilicus [29]. Only one of the four patients who presented with TSH had off-midline trocar placement, as in our case; however, the patient was 79 years old, which was a risk factor for TSH. Considering all of this information, the exact cause of the TSH in our case remains unknown because the trocar was not placed in the midline, SILS was not performed, and the patient was not an older adult. However, there was still the possibility that excessive manipulation, such as stretching of the port site when removing the specimen, resulted in the TSH.

We have previously reported that the surgical specimen could be removed via transvaginal route instead of extraction from the trocar site since the adenomyotic tissue can be too tough to extract using the morcellator from the 12 mm trocar [30, 31]. In addition, some surgeons also opted for vaginal extraction due to improved cosmetic outcomes in some gynecologic laparoscopic surgeries [32–34]. Recently, Huang et al. proposed devised shape incision, such as Y-shape, to extract the specimen using an animal model. This technique could reduce the length of incision and using this technique clinically could minimize complications associated with auxiliary incision, such as TSH [35]. All the above points make a strong argument for using vaginal extraction route in patients with risk for TSH.

**Fig. 7:**
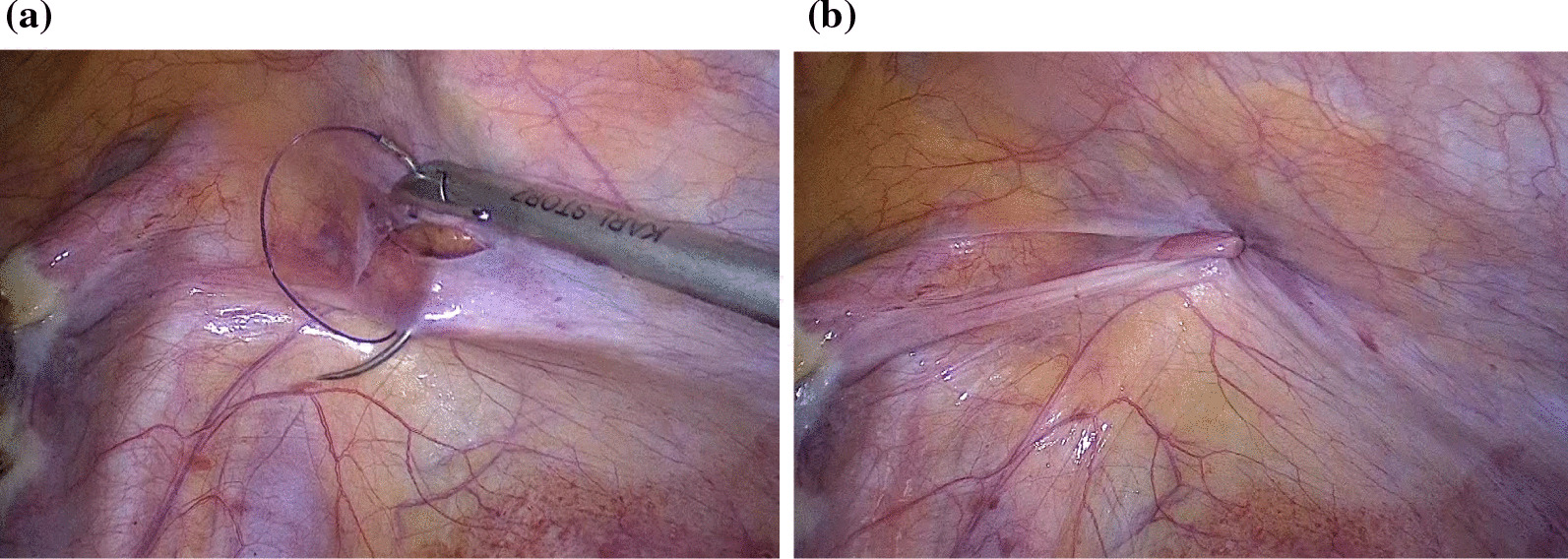
**a**, **b** Once the laparoscopic procedure was completed, the peritoneum was laparoscopically closed using a Z suture with 2-0 polydioxanone

Several surgeons have introduced a method of fascial closure that uses a new device and technique to prevent TSH [14, 21, 36]. Although immediate postoperative pain has been prevented with the use of local anesthetics, such as bupivacaine, at port sites during surgery, postoperative abdominal pain still occurred at the fascial closure site due to the fascial edge bridging, excessive fascial tension, and potential nerve entrapment [37]. Therefore, we were hesitant to suture the fascia and only sutured the peritoneum with 2-0 PDS under direct vision while maintaining a pneumoperitoneum (Fig. 7a, b; Additional file 1: Movie). The procedure was performed in this manner because studies have found that TSH can develop below the fascia; therefore, peritoneal closure is more important than fascial closure [14]. To date, this method has been used in approximately 50 patients with no complications or recurrence. Further cases should be documented, and these patients should be followed up to determine the long-term prognosis after using this surgical technique.

In conclusion, we understand that TSH is a relatively uncommon complication of gynecologic laparoscopy. In 40 years of experience, despite improved equipment and more skilled gynecologists, this was our first encounter with TSH. Greater attention should be paid to the possibility of TSH to ensure the prevention severe problems through early detection and treatment.

## Supplementary Information

**Additional file 1:** Movie.

## Data Availability

The data that support the findings of this study are available from the corresponding author, KO, upon reasonable request.

## Abbreviations

TSH: Trocar site hernia
BMI: Body mass index
CO_2_: Carbon dioxide
SILS: Single-incision laparoscopic surgery
USA: United States of America
